# SV40 Utilizes ATM Kinase Activity to Prevent Non-homologous End Joining of Broken Viral DNA Replication Products

**DOI:** 10.1371/journal.ppat.1004536

**Published:** 2014-12-04

**Authors:** Gregory A. Sowd, Dviti Mody, Joshua Eggold, David Cortez, Katherine L. Friedman, Ellen Fanning

**Affiliations:** 1 Department of Biological Sciences, Vanderbilt University, Nashville, Tennessee, United States of America; 2 Department of Biochemistry, Vanderbilt University School of Medicine, Nashville, Tennessee, United States of America; University of Wisconsin-Madison, United States of America

## Abstract

Simian virus 40 (SV40) and cellular DNA replication rely on host ATM and ATR DNA damage signaling kinases to facilitate DNA repair and elicit cell cycle arrest following DNA damage. During SV40 DNA replication, ATM kinase activity prevents concatemerization of the viral genome whereas ATR activity prevents accumulation of aberrant genomes resulting from breakage of a moving replication fork as it converges with a stalled fork. However, the repair pathways that ATM and ATR orchestrate to prevent these aberrant SV40 DNA replication products are unclear. Using two-dimensional gel electrophoresis and Southern blotting, we show that ATR kinase activity, but not DNA-PK_cs_ kinase activity, facilitates some aspects of double strand break (DSB) repair when ATM is inhibited during SV40 infection. To clarify which repair factors associate with viral DNA replication centers, we examined the localization of DSB repair proteins in response to SV40 infection. Under normal conditions, viral replication centers exclusively associate with homology-directed repair (HDR) and do not colocalize with non-homologous end joining (NHEJ) factors. Following ATM inhibition, but not ATR inhibition, activated DNA-PK_cs_ and KU70/80 accumulate at the viral replication centers while CtIP and BLM, proteins that initiate 5′ to 3′ end resection during HDR, become undetectable. Similar to what has been observed during cellular DSB repair in S phase, these data suggest that ATM kinase influences DSB repair pathway choice by preventing the recruitment of NHEJ factors to replicating viral DNA. These data may explain how ATM prevents concatemerization of the viral genome and promotes viral propagation. We suggest that inhibitors of DNA damage signaling and DNA repair could be used during infection to disrupt productive viral DNA replication.

## Author Summary

Viruses from both *Polyomaviridae* and *Papillomaviridae* families share several characteristics. These include common modes of DNA replication and an accumulation of DNA damage signaling and repair proteins at replicating viral DNA. Several DNA repair proteins, with unknown functions during viral DNA replication, associate with the viral replication centers of the polyomavirus simian virus 40 (SV40). In this study we examined the mechanisms that regulate and recruit DNA repair machinery to replicating viral DNA during permissive SV40 infection. We found that the virus deploys DNA repair to broken viral DNA using cellular DNA damage signaling pathways. Our results shed light on why both *Polyomaviridae* and *Papillomaviridae* DNA replication elicits DNA damage signaling and repair. As no effective treatments currently exist for the *Polyomaviridae* family, our data identify pathways that might be therapeutically targeted to inhibit productive viral replication. Additionally, we categorize distinct functions for DNA repair and damage signaling pathways during viral replication. The results provide insights into how viruses exploit cellular processes to overwhelm the cell and propagate.

## Introduction

A diverse set of protein functions is required to ensure the timely, accurate duplication of the genome. In addition to the components of the replication machinery itself [1], [2], accurate replication requires the S phase checkpoint kinase, ataxia telangiectasia-mutated and rad3-related (ATR). ATR and its related kinases, ataxia telangiectasia-mutated (ATM) and DNA-protein kinase catalytic subunit (DNA-PK_cs_), are members of the PI3K-related protein kinase (PIKK) family that regulate DNA damage signaling in response to various endogenous and exogenous stresses [3]. ATR kinase function is primarily activated by DNA replication stress through the capacity of the ATR/ATRIP complex to sense stretches of replication protein A (RPA)-bound single-stranded DNA [4]. ATM and DNA-PK_cs_ function to promote DNA repair and are primarily activated in response to double strand breaks (DSB) [3]. To identify DSBs, ATM and DNA-PK_cs_ rely on MRE11-RAD50-NBS1 (MRN) and KU70/80 (KU), respectively [5]. DNA-PK_cs_ promotes non-homologous end joining (NHEJ) [6]. On the other hand, either ATM- or ATR-dependent phosphorylation events are accompanied by activation and recruitment of numerous factors that influence DNA repair and mediate arrest of both the cell cycle and DNA replication [3].

Several DNA repair proteins are required for the successful completion of cellular DNA replication, particularly those of the homology-directed repair (HDR) pathway. HDR is initiated by MRN recognition of DSB termini [7]. The S phase specific interaction of MRN with CtIP, a processivity factor for the MRE11 nuclease [8], [9], enables the initiation of 5′ to 3′ end resection to create a short 3′ tail. The recessed 5′ end can be subsequently digested by the more processive nucleases EXO1 and the BLM/DNA2 complex [10] to generate a lengthy 3′ tail that can be bound by the RAD51 recombinase to catalyze strand invasion and displacement loop formation [11], [12]. HDR is commonly activated following replication stress or by agents that elicit DSBs in S and G2 phases [13]. Inactivation of HDR factors causes slowed DNA synthesis [14], instability of nascent DNA strands [15], anaphase bridges owing to un-replicated DNA entering mitosis [16], [17], [18], and increased genome breakage in both the presence and absence of replication stress [19], [20], [21]. Several of these characteristics are reminiscent of the defects observed in Seckel syndrome cells, which harbor hypomorphic mutations in the ATR or CtIP genes, providing a possible link between HDR and DNA damage signaling [22], [23], [24], [25]. Thus, both ATR kinase signaling and HDR functions are required during S phase to promote genome stability.

Similar to studies of cellular DNA replication, recent evidence suggests that polyomaviruses and papillomaviruses might utilize a more extensive set of DNA replication factors than previously anticipated [26], [27], [28], [29], [30]. Infection of cells by polyomaviruses and papillomaviruses is accompanied by intense ATM and ATR activation [26], [28], [31], [32], [33]. However, viral DNA replication continues despite DNA damage checkpoint signaling (for review see [34]). ATM and ATR kinase activities facilitate the repair of replication-associated breaks on viral chromatin during SV40 DNA replication and failure to repair these breaks results in the accumulation of concatemers of the SV40 genome through rolling circle replication [30]. On the other hand, DNA-PK_cs_ kinase activity is not required for normal viral replication [30]. SV40 has been used to identify and characterize numerous cellular DNA replication factors that together with the viral origin recognition complex/replicative DNA helicase, large T antigen (Tag), can replicate the viral genome [1], [34]. Thus, DNA damage signaling and repair proteins used by SV40 DNA replication might represent a set of factors employed during normal cellular DNA replication to prevent and/or repair replication-associated DSBs.

Here we show that, in addition to rolling circle formation, inhibition of ATM or ATR causes the accumulation of broken, non-replicating linear viral DNA. We examined which repair proteins co-localize with sites of viral replication during unperturbed infection to define further the cellular DNA repair factors that associate with replicating SV40 chromatin and contribute to viral replication. We find that HDR activities are strongly recruited to Tag foci, but NEHJ factors are not. The selective recruitment of HDR factors and exclusion of NHEJ proteins requires ATM but not ATR kinase activity. Although ATM and ATR contribute to cell cycle arrest in infected cells, the activity of ATM in regulating DSB repair choice is independent of its cell cycle checkpoint function. Our results suggest that while ATM and ATR kinase inhibition results in the accumulation of multiple forms of broken viral DNA, DNA-PK_cs_ function at replicating viral DNA is limited to instances when ATM kinase activity is inhibited. Our results lend further insight into how SV40 manipulates the DNA damage response to promote its replication inside infected cells.

## Results

### ATM and ATR cooperate to prevent the accumulation of DNA DSBs during SV40 DNA replication

**Figure 1:**
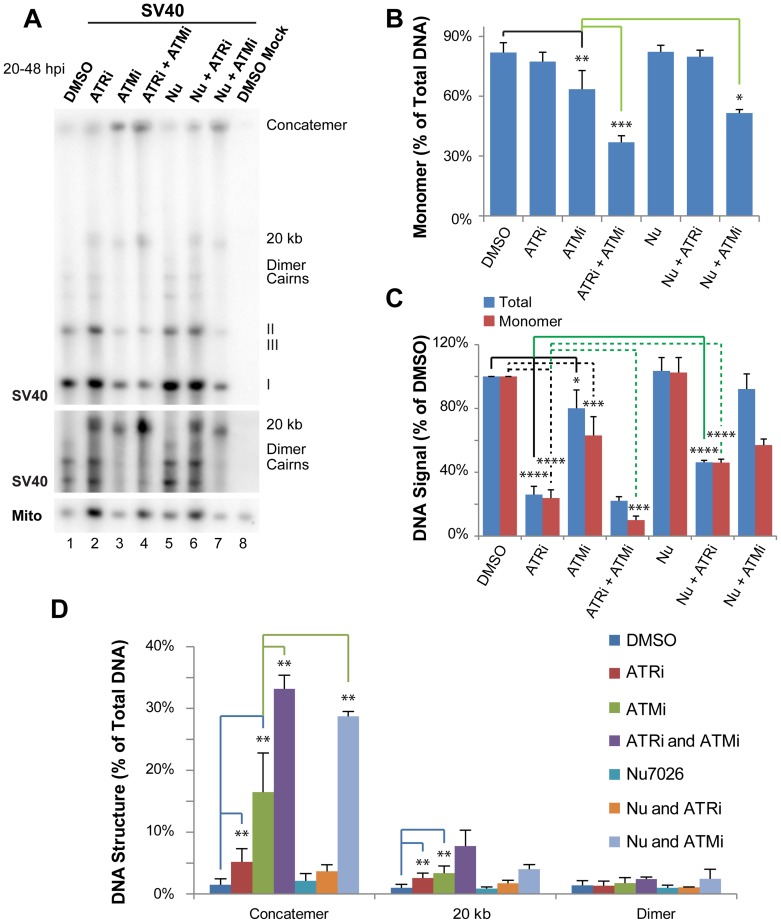
ATR and DNA-PK_cs_ prevent viral genome concatemer formation when ATM is inhibited. (A) Southern blot of DNA extracted from SV40-infected BSC40 cells treated with combinations of DMSO, ATRi, ATMi, and/or Nu7026 during the final 28 h of a 48 h infection. The middle panel shows a longer exposure of a portion of the Southern blot pictured in the top panel. To emphasize SV40 replication intermediates and aberrant products, equal amounts of total SV40 DNA were loaded into each lane. (B) Quantification of monomer accumulation when PIKK(s) are inhibited from Southern blots as shown in (A). (C) Graph of total viral or SV40 monomer DNA signals normalized to SV40 DNA replicated in the presence of DMSO from Southern blots as shown in (A). (D) Graph of aberrant structure(s) accumulated as a result of single or multiple PIKK inhibition from Southern blots as shown in (A). In (B–D), bars for DMSO, ATRi, and ATMi show the average of 6 to 7 independent experiments. In the same panels, the bar for Nu-7026 shows the average of 4 independent experiments; whereas bars for combinations of inhibitors (ATMi/ATRi, Nu7026/ATRi, and ATMi/Nu7026) show the average of 3 independent experiments.

We previously observed that inhibition of either ATM or ATR caused increased accumulation of both broken unidirectional replication forks and head to tail repeats of the viral genome known as concatemers [30]. The topological status of aberrant viral DNA intermediates and products accumulating when ATM or ATR were inhibited was not examined in these studies nor was the potential redundancy between the two kinases established. To examine viral replication products in detail, DNA was extracted at 48 hours post infection (hpi) from cells treated with ATRi (ATR inhibitor VE-821 [35]), ATMi (ATM inhibitor Ku-55933 [36]), both ATRi and ATMi, or the solvent DMSO from 20 to 48 hpi. DNA was digested with *Xba*I and *Sac*I, enzymes that cleave frequently within genomic DNA but for which recognition sites are absent in the SV40 viral genome. As previously reported, one-dimensional (1D) agarose gel electrophoresis followed by Southern blotting and hybridization with a probe that recognizes the viral genome primarily detected unit length, monomeric viral genomes of form I (supercoiled) and form II (nicked), with very little monomer form III (linear; Figure 1A, lane 1 and 1B). Upon longer exposure, slower migrating species could be detected that correspond to circular intermediates of bidirectional replication (late Cairns structures [37]) and dimeric genomes [38], [39] (Figure 1A, bottom panel).The total amount of viral DNA produced during infection was quantified using a probe for mitochondrial DNA as an internal standard.

Inhibition of either ATM or ATR alone significantly decreased viral DNA yield as compared with DMSO-treated cells, although the effect was more pronounced for ATRi, as previously reported [30] (Figure 1C). As expected from our previous work [30], treatment with either ATMi or ATRi increased the percent of total viral DNA that migrated at an apparent size of ∼20 kb or failed to enter the gel (Figure 1A, D) and significantly decreased the fraction of viral DNA in monomer form (ATMi treatment only; Figure 1B). We have previously demonstrated that the viral DNA that remains in the well consists of concatemers of the viral genome produced by rolling circle replication (30), however the nature of the product running at an apparent size of ∼20 kb was not previously addressed.

To examine functional overlap between ATM and ATR, cells were treated simultaneously with both inhibitors. While this treatment did not significantly decrease the amount of total viral DNA produced compared to ATR inhibition alone (Figure 1C), the production of unit length monomer was synergistically decreased compared to either single treatment (Figure 1A, B). This defect in form I, II, and III monomer production when ATM and ATR were both inhibited was accompanied by a 2 and 2.3 fold increase in the accumulation of concatemers and the ∼20 kb products, respectively, compared to ATM inhibition alone (Figure 1A, D). This result suggests that ATM and ATR kinase activities are partially redundant in their ability to prevent concatemer and other aberrant product formation during SV40 replication.

**Figure 2:**
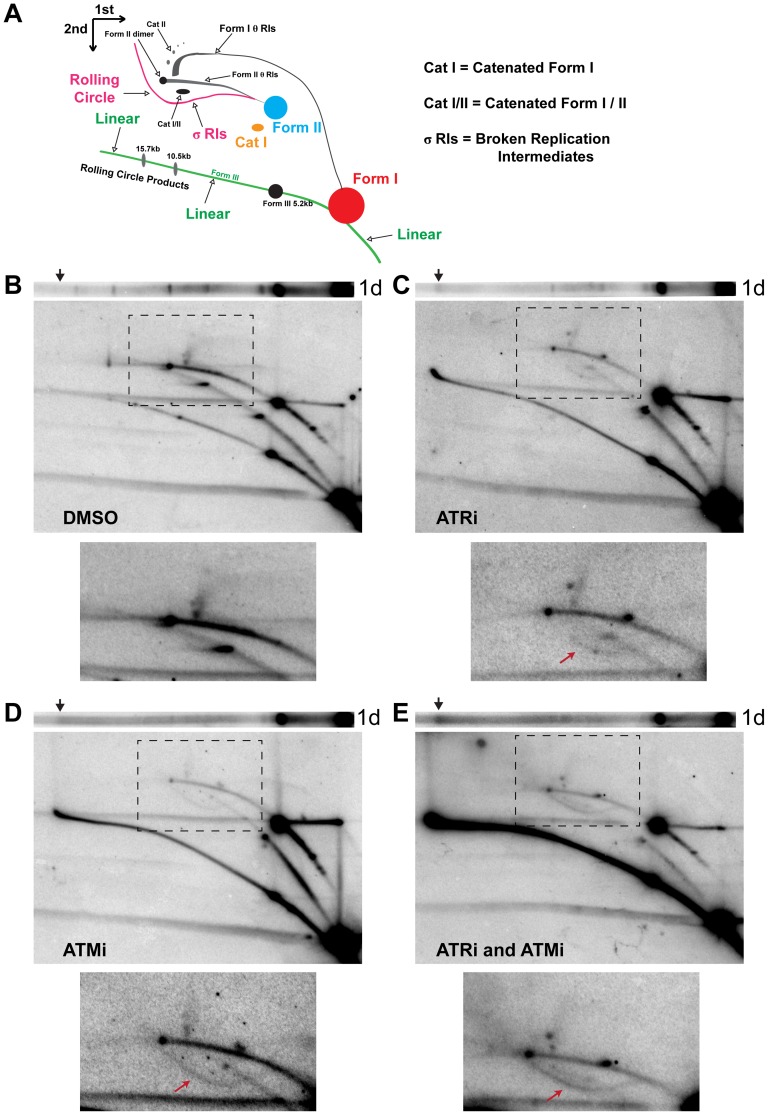
ATM or ATR inhibition increases broken replication forks and linear viral DNA replication products. (A) Diagram of 2D gel electrophoresis of undigested circular dsDNA [86]. (B, C, D, E) Southern blots of the first dimension of a neutral 1D gel (top panel) or 2D gel (middle panel) from SV40-infected BSC40 cells exposed to DMSO (B), ATRi (C), ATMi (D), or ATRi and ATMi (E) during the last 28 h of a 48 h SV40 infection. Arrow on 1D gel points to the location of the ∼20 kb replication product. Bottom panel: Enlargement of the picture within the boxed area in middle panel. Exposure of the bottom panel was increased to enhance visualization of θ and σ replication intermediates shown in (A). Arrow in lower panels points to location of the σ arc.

To gain insight into the topological status of the observed aberrant products, the DNAs described above were subjected to two-dimensional (2D) agarose gel electrophoresis and Southern blotting (Figure 2). This method can resolve circular (form I and form II) and linear (form III) viral DNA replication products from intact or broken viral replication intermediates and large, unbranched linear forms of the viral genome (Figure 2A) [40], [41]. Analysis of the undigested viral DNA from DMSO-treated SV40-infected cells by 2D gel electrophoresis demonstrated that the majority of viral replication intermediates consisted of intact form II theta molecules (Figure 2B), as expected from the divergence of two forks from a single viral origin of replication. The majority of non-replicating viral DNAs were form I or II monomers (Figure 2B). When infected cells were exposed to ATRi, there was a pronounced increase in the amount of linear viral DNA at greater-than-monomer length (compare Figure 2B and C, linear arc). Furthermore, a partial sigma arc (Figure 2A; pink line marked “rolling circle”) was present upon ATRi treatment and was absent from DNA extracted from DMSO-treated cells (Figure 2B, C, lower panel, arrow). Sigma forms are generated from breakage of one replication fork in theta form DNA, followed by fork arrest or unidirectional, rolling circle replication. In ATRi-treated cells, virtually all viral DNA that migrated at ∼20 kb in the first gel dimension (Figure 2C, arrow) migrated as expected for linear molecules in the second dimension. When infected cells were exposed to ATMi, both intense linear and full sigma arcs were present (Figure 2D, arrow points to sigma arc), consistent with high levels of viral DNA breakage. Notably, the spots corresponding to nicked, catenated, and head to tail dimer intermediates were greatly decreased by ATM inhibition relative to DMSO treatment (Figure 2D).

As predicted from the 1D gel analysis, combined inhibition of ATR and ATM increased all broken forms of viral DNA relative to DMSO, ATRi, or ATMi treated cells (Figure 2E, linear and sigma arcs). The prominent sigma arc, corresponding to unidirectional replication, correlated with a great decrease in fork convergence products (Cat I, CatI/II, and Dimer; Figure 2E, lower panel). Additionally, the ∼20 kb product detected in the first dimension (Figure 2E, arrow) again migrated on the linear arc of the 2D gel (Figure 2E). We conclude that the products migrating at an apparent size of ∼20 kb are aberrant linear forms of the viral genome running at the limiting mobility of the gel. These molecules most likely result from breakage of a rolling circle intermediate during SV40 replication. Taken together, these results suggest that ATM and ATR function to prevent DSB accumulation during viral replication and that each kinase is able to partially substitute for the other in this function.

### DNA-PK_cs_ contributes to viral replication fork repair when ATM is inhibited

DNA-PK_cs_ is robustly activated when ATM or ATR are inhibited during SV40 DNA replication [30]. Thus, the NHEJ pathway of DSB repair directed by DNA-PK_cs_ might fulfill some aspect of DNA repair upon ATM or ATR inhibition during SV40 DNA replication. To examine this possibility, SV40-infected cells were treated with a combination the DNA-PK_cs_ inhibitor Nu7026 [42], [43] and DMSO, ATMi, or ATRi from 20 to 48 hpi. DNA was extracted at 48 hpi, separated in a single dimension by gel electrophoresis, and subjected to Southern blotting (Figure 1A). DNA-PK_cs_ inhibition alone with Nu7026 had no effect on any aspect of SV40 DNA replication (Figure 1A–D) [30]. Likewise, inhibition of DNA-PK_cs_ in combination with ATR inhibition did not increase the appearance of aberrant products relative to ATR inhibition alone (Figure 1A, D). Indeed, DNA-PK_cs_ inhibition partially rescued the reduction in total viral replication levels observed upon ATR inhibition alone (Figure 1C). We conclude that DNA-PK_cs_ is unlikely to contribute to the repair of DSBs formed when ATR kinase activity is blocked, but may contribute to the inhibition of viral replication observed in the absence of ATR function.

Simultaneous exposure of cells to ATMi and Nu7026 had only a minimal effect on overall levels of viral DNA replication (Figure 1A, C). However, the percentage of monomeric products decreased from 64% when ATM was solely inhibited to 50% when ATM and DNA-PK_cs_ were inhibited (Figure 1A, B). Concomitant with the decrease in total monomer fraction, the relative fraction of concatemeric forms increased 1.7 fold relative to ATM inhibition alone (Figure 1A, D). These data imply that NHEJ orchestrated by DNA-PK_cs_ may facilitate the repair of a portion of DSBs formed when ATM is inhibited. Alternatively, the inhibited DNA-PK_cs_ bound to DSBs that accumulate upon ATM inhibition could create a barrier to DSB repair by preventing access of DSB termini to 5′ to 3′ end resection proteins [44], [45], [46].

### Homology-directed repair factors colocalize with viral replication centers

The pronounced increase in viral replication products indicative of unrepaired DSB observed upon inhibition of ATR and/or ATM suggested that normal viral replication may require the function of DSB repair factors. Several such factors with poorly understood functions in SV40-infected cells have been found to concentrate at or near SV40 DNA replication centers [26], [27]. Many of these factors, including MRN, RAD51, FANCD2, and BRCA1 function in HDR [3] and might act to repair broken DNA resulting from viral DNA replication [30]. To define further which DSB repair activities are recruited to or localized near viral DNA replication centers, the localization of chromatin-bound DSB repair proteins relative to Tag foci was examined in SV40-infected BSC40 and U2OS cells using fluorescence microscopy.

**Figure 3:**
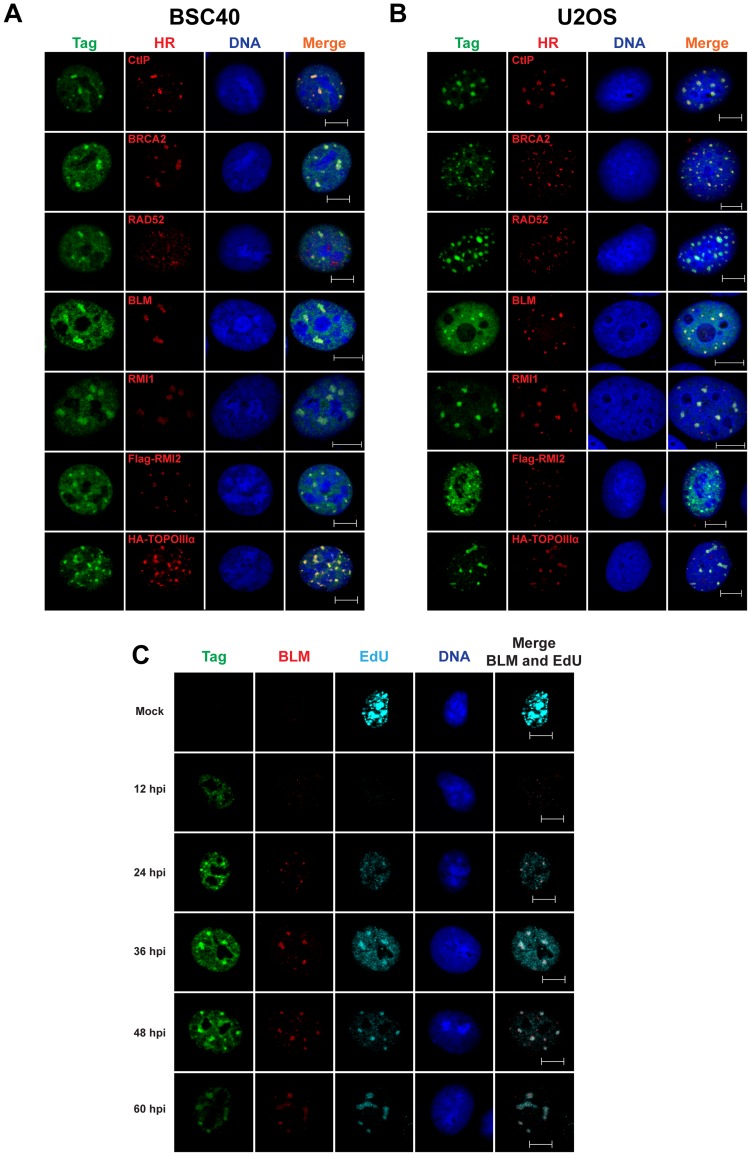
SV40 DNA replication centers colocalize with HDR proteins. (A–C) Images of chromatin-bound Tag and the indicated HDR factors from SV40-infected BSC40 (A, C) or U2OS (B) cells at 48 hours post infection. Vectors for expression of HA-topoisomerase IIIα or Flag-RMI2 were transfected 24 h prior to infection. In (C), 20 µM EdU was present in the media of SV40-infected BSC40 cells during the final 5 minutes prior to fixation. Scale bar in all images represents 10 µm.

CtIP, a protein critical for HDR due to its role in the initiation of 5′ end resection [8], formed intense foci that colocalized with Tag in SV40-infected BSC40 and U2OS cells (Figure 3A, B). Furthermore, the RAD51 loader BRCA2, the single-strand annealing protein RAD52, and the dHJ dissolvase BTR (BLM, RMI1/2, and Topoisomerase IIIα) colocalized on chromatin with viral DNA replication centers in infected BSC40 and U2OS cells (Figure 3A, B). If these proteins are needed for the completion of normal viral replication, the kinetics with which repair factors arrive at the viral replication center should correlate with the time of DNA replication. To address this possibility, the nuclear dynamics of the BTR component BLM were examined during a 60 h time course of SV40 infection in BSC40 cells. The colocalization of BLM with viral DNA replication centers was greatest at times longer than 24 hpi (Figure 3C). This timeframe of BLM colocalization with Tag correlated well with the reported timing of viral DNA replication [47], [48] and with the observed incorporation of EdU at Tag foci (Figure 3C). The colocalization of BLM and Tag is reminiscent of that found for MRN and Tag in SV40-infected cells [26]. These results suggest that localization of HDR to the viral DNA replication centers marked with Tag is a characteristic of SV40-infected cells.

### NHEJ proteins do not localize to Tag foci in SV40-infected cells

**Figure 4:**
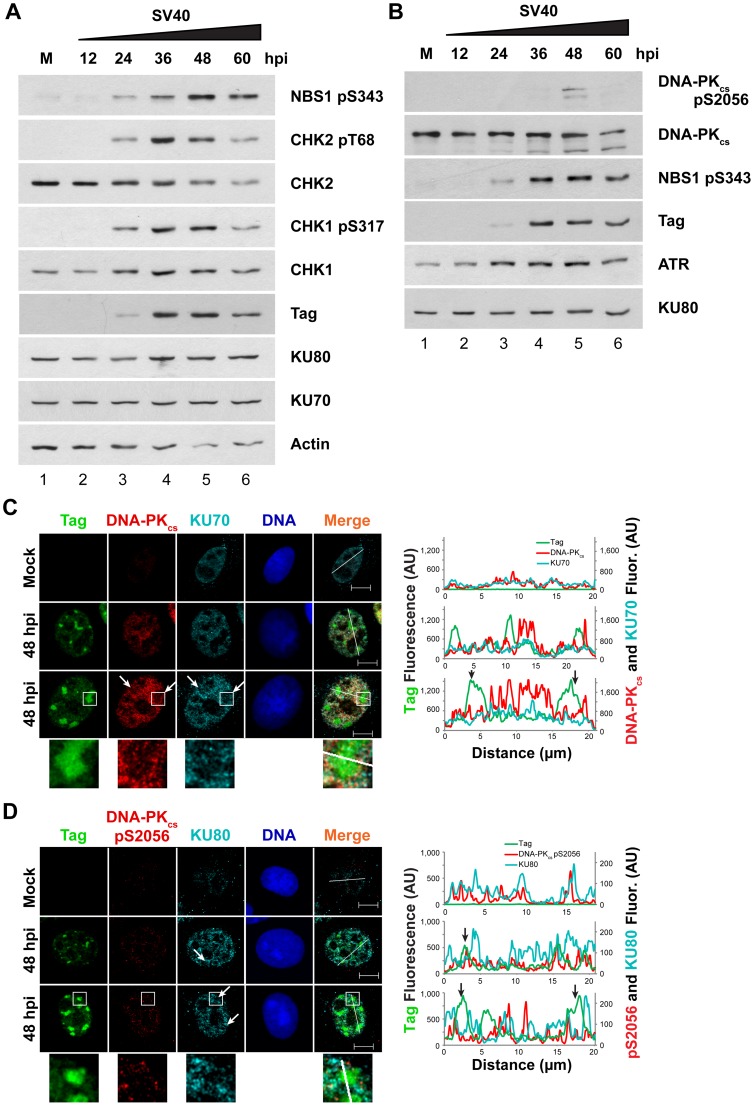
Factors that promote NHEJ do not co-localize with Tag in SV40-infected BSC40 cells. (A, B) Western blot of cell lysates from SV40- or mock (M)-infected BSC40 cells at the indicated timepoints. The two blots are from the same lysate isolated from one representative experiment and each blot was re-probed for the indicated proteins. (C, D) Representative pictures of chromatin-bound Tag or DNA-PK from SV40- or mock-infected BSC40 cells at 48 hpi. Merged images show DNA-PK_cs_, KU, and Tag. Bottom panel of (C) and (D) shows an enlargement of the boxed area. The fluorescence intensity in arbitrary units (AU) along the line shown in the merged image is graphed in the right panel. Arrows point to a region on the line drawing at which decreased signal of DNA-PK_cs_ or KU at the replication center is observed. Scale bars represent 10 µm.

Similar to HDR protein recruitment to viral replication centers, SV40 infection induces ATM and ATR DNA damage signaling in a time frame that closely mirrors viral DNA replication (Figure 4A). Thus, a related PIKK, DNA-PK_cs_, might be activated with similar kinetics during SV40 infection. To assess DNA-PK_cs_ kinase activation and the total DNA-PK_cs_, KU70, and KU80 protein levels during viral infection, steady-state levels of these proteins were determined by immunoblotting in extracts of SV40-infected BSC40 cells harvested over a 60 h time course (Figure 4A, B). These blots revealed that ATR and ATM began to phosphorylate substrates CHK1 and CHK2, respectively, at 24 hpi (Figure 4A). ATM and ATR activities were greatest from 36 through 48 hpi, after which they began to decline (Figure 4A, compare lanes 4, 5 to lane 6). A second marker of ATM activation, NBS1 pS343, displayed similar kinetics of phosphorylation, although it may peak slightly later than CHK2 pT68 (Figure 4A, B). On the other hand, DNA-PK_cs_ kinase activation was observed most strongly at 48 hpi and more weakly at 36 and 60 hpi, as assessed by DNA-PK_cs_ autophosphorylation at S2056 (Figure 4B, compare lane 1 to lanes 4–6) [44], [49], [50], [51], [52]. The total protein levels of KU70, KU80, and DNA-PK_cs_ were stable throughout 60 h of SV40 infection (Figure 4A, B, compare lanes 1–6). Thus, the timing of DNA-PK_cs_ kinase activation during SV40 infection poorly correlates with viral DNA replication.

To determine whether the limited DNA-PK_cs_ kinase activation observed at 48 hpi was associated with localization of the DNA-PK holoenzyme to viral DNA replication centers, chromatin-bound NHEJ proteins KU70, KU80, and DNA-PK_cs_ were examined by fluorescence microscopy at 48 hpi in SV40-infected BSC40 and U2OS cells. DNA-PK_cs_, KU70, and KU80 showed little preference for binding at or near viral DNA replication centers in both BSC40 and U2OS cells (Figures 4C, D and S1). DNA-PK_cs_ auto-phosphorylated on S2056 was not abundant on the chromatin of SV40-infected cells at 48 hpi and did not associate with viral DNA replication centers (Figures 4D and S1B). Indeed, close inspection of viral replication centers revealed that DNA-PK_cs_ and KU70/80 appeared to be excluded from Tag foci (Figures 4C, D and S1, arrows, enlarged boxes). Line tracings through viral replication centers confirmed that intense KU70/80 and DNA-PK_cs_ foci did not colocalize with Tag (Figures 4C, D and S1). Taken together, these results are consistent with a correlation between ATM, ATR, and HDR activation and the timing of viral DNA replication. In contrast, DNA-PK_cs_ activation correlates poorly with viral replication.

### ATM and ATR contribute differentially to S phase arrest of SV40 infected cells

**Figure 5:**
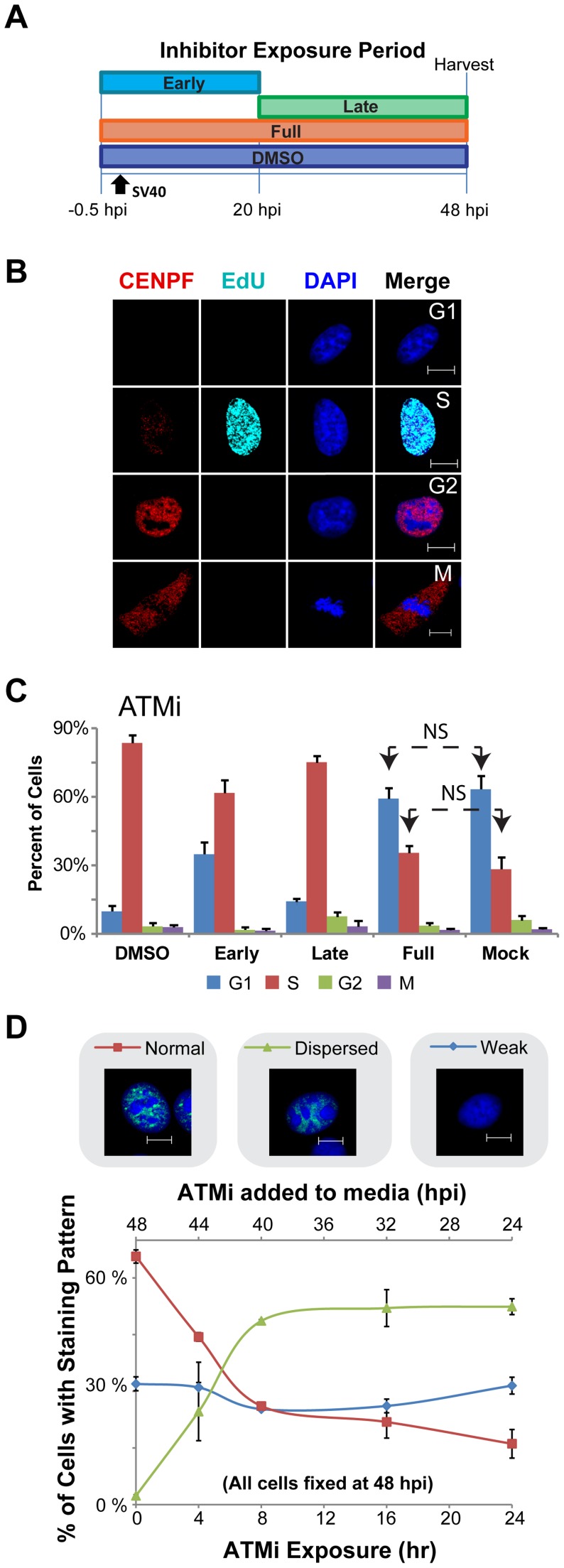
ATM contributes to S phase arrest during SV40 infection in BSC40 cells. (A) Experimental scheme for treatment of cells with ATMi during phases of a 48 h SV40 infection. Early: inhibitor present from −0.5 to 20 hpi. Late: inhibitor present from 20 to 48 hpi. DMSO and Full: solvent or inhibitor, respectively, present from −0.5 to 48 hpi. (B) Representative images of fixed and then permeablized BSC40 cells. Prior to fixation, cells were incubated for 10 minutes with 20 µM EdU. Scale bar represents 10 µm. Cells in G1 do not stain for CENPF and do not contain EdU pulse label. S phase cells have EdU labeled DNA, whereas G2 cells lack EdU pulse label, but contain nuclear CENPF. Mitotic cells have cytoplasmic CENPF and condensed chromatin. (C) Graphs of the stage of the cell cycle of cells exposed to ATMi as in (A). Cell cycle stage was determined as described in (B). In (C), all G1 and S bars are significantly different (p<0.05 by two tailed student's t test) than the corresponding bars in DMSO or Mock controls except those denoted NS (not significant). Error bars represent standard deviation. Graphs show the average of 3 independent experiments. (D) Graph of Tag staining patterns of SV40-infected BSC40 cells treated with ATMi for the last 0, 4, 8, 16, 24 h of a 48 h SV40 infection. Micrograph shows an example of each chromatin-bound Tag staining pattern. No points on the “weak Tag staining pattern” line are significantly different from SV40 infected cells treated with DMSO during the final 24 h of a 48 h infection (0 h ATMi). All data points on the “dispersed” and “normal staining pattern” lines are significantly different than the 0 h ATMi control (p<0.05). Each data point on the graph represents the average of 3 independent experiments. Error bars show standard deviation.

Having characterized the DNA repair factors that localize at viral replication centers during SV40 infection, we were interested in determining whether ATM and ATR are required for that recruitment. However, ATM kinase activity is known to contribute both to cell cycle arrest and to DNA repair efficiency after DNA damage [3], [53]. ATM inhibition increases aberrant SV40 DNA replication products (Figures 1, 2, and see reference [30]) and results in a dispersed, non-focal Tag staining pattern [26], [30], but the relative contribution of different ATM functions to these phenotypes is unclear. We sought to separate these functions of ATM by varying the time period of viral infection during which ATM inhibitor was applied. SV40-infected BSC40 cells were exposed to ATMi during an early period (from 30 minutes prior to infection through 20 hpi), a late period (from 20 to 48 hpi), or for the duration of the infection (Figure 5A) [30]. The early phase corresponds to the cellular G1 to S transition and viral early gene expression while the late phase encompasses the period of viral DNA replication (Figure 5A). SV40-infected cells exposed to the inhibitor solvent, DMSO, served as a positive control for S phase arrest, and uninfected cells were used to determine cell cycle distribution in an asynchronous population of cells (Figure 5A). Cell cycle stage was determined by measuring the presence of CENPF immunostaining and EdU incorporation into DNA (Figure 5B) [54], [55], [56].

As expected, a substantial percentage of mock-infected cells were in G1 and S phase (Figure 5C). Upon SV40 infection in the presence of DMSO, ∼90% of SV40-infected BSC40 cells arrested in S phase, consistent with checkpoint activation (Figure 5C). Conversely, similar percentages of G1 and S phase cells to those observed in asynchronous, mock-infected BSC40 cells were observed when ATM was inhibited for the duration of infection (Figure 5C). The presence of ATMi during the early phase of infection partially relieved the S phase arrest (Figure 5C). In contrast, inhibition of ATM during the late phase of infection only very weakly attenuated the SV40-induced S phase arrest (Figure 5C). Taken together, the results suggest that the ATM-CHK2 pathway is required during the early phase of infection for efficient S phase arrest.

To determine the minimum time of exposure to ATMi required to induce the characteristic non-focal viral replication centers to form, the chromatin-bound Tag staining pattern was monitored by immunofluorescence microscopy from infected cells when ATMi was added to the medium during the final 4, 8, 16, or 24 h of a 48 h infection. DMSO-treated, SV40-infected cells demonstrated the intense Tag foci that normally accompany productive infection, with a smaller percentage of cells showing a weak staining pattern that lacked chromatin-bound Tag (Figure 5D, 0 h ATMi exposure). The addition of ATMi for 4 h prior to the 48 hpi fixation time point caused the appearance of cells showing an aberrant, dispersed Tag staining pattern (23%; Figure 5D). Incubations equal to or greater than 8 h with ATMi increased the number of cells showing this dispersed Tag staining pattern to ∼49% (Figure 5D). During the time period tested by this experiment, the prevalence of the weak staining pattern was unaffected by the presence of ATMi (Figure 5D). This weak pattern may represent cells that failed to arrest in S phase. Since an 8 h ATMi exposure is sufficient to stably produce aberrant Tag immunostaining, while no effect on the infected cell cycle is observed with ATM inhibition during this time period, an 8 h exposure to ATMi was utilized in subsequent experiments.

Similar to ATM, ATR also modulates DNA repair and cell cycle checkpoint control during S phase [3]. To determine the effect of ATR kinase activity on cell cycle arrest in infected cells, SV40-infected BSC40 cells were exposed to ATRi during periods of SV40 infection as shown in Figure S2A, pulsed with EdU, fixed and immunostained for CENPF to determine the phase of the cell cycle. Inhibition of ATR during any phase of infection compromised cell cycle arrest as indicated by an increased percentage of cells in G1 phase and a decreased percentage of cells in S phase (Figure S2B). In contrast to ATMi treatment, ATRi treatment during the last 8 hours of the 48-hour infection did not induce the formation of the dispersed Tag staining pattern. However, the number of cells showing a weak Tag pattern increased from ∼32% in the DMSO-treated infected control to ∼58% after ATR inhibition (Figure S2C). Only ∼14% of cells with the weak Tag staining pattern incorporated EdU, while ∼96% of the ATRi-treated cells with normal Tag staining pattern showed EdU incorporation (Figure S2C). This result aligns with the observation that the presence of ATRi from 40 to 48 hpi decreased the fraction of cells in S phase from 87% in the DMSO control infection to 46% (Figure S2B). Again, we attribute the increase in “weak” Tag staining cells to those that have escaped S phase arrest. Cells that do remain in S phase appear to retain normal morphology of the viral replication center as measured by Tag staining (Figure S2C). We conclude that ATR contributes to S phase arrest of SV40 infected cells throughout infection, but does not affect viral replication center stability in the fraction of cells that remain arrested in S phase.

**Figure 6:**
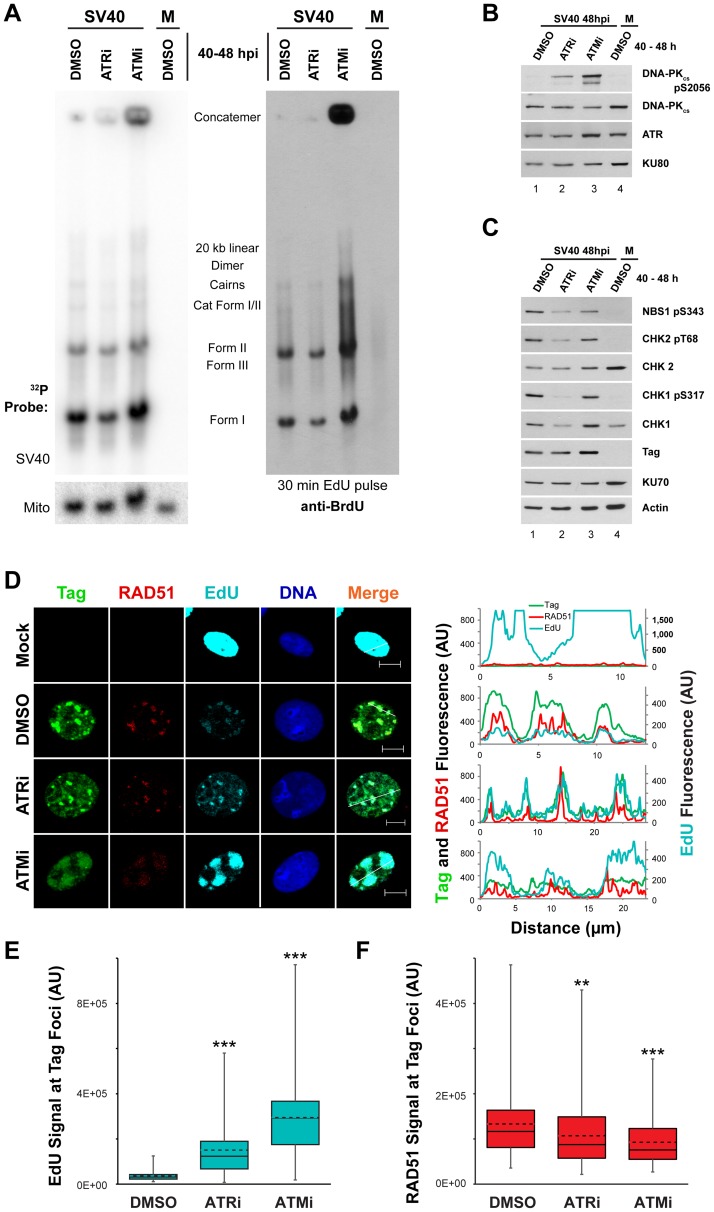
ATM or ATR inhibition does not affect incorporation of nucleotides into SV40 DNA. (A) Southern blot of DNA extracted from SV40-infected BSC40 cells treated with DMSO, ATRi, or ATMi from 40 to 48 hpi and labeled with 20 µM EdU for 30 minutes prior to DNA extraction. Left Panel: phosphorimager scan of Southern blot probed for mitochondrial or SV40 DNA. Right Panel: same Southern blot as in left panel but probed for EdU with an anti-BrdU antibody. (B, C) Western blots of cells lysates from SV40-infected BSC40 cells treated with DMSO, ATRi, or ATMi during the final 8 h of a 48 h SV40 infection. (D) Immunofluorescence microscopy of the indicated factors from SV40- or mock-infected BSC40 cells treated with DMSO, ATRi, or ATMi during the final 8 h of a 48 h SV40 infection. Prior to fixation at 48 hpi, cells were pulsed with 20 µM EdU for 5 minutes and non-chromatin bound proteins were extracted from cells. The fluorescence intensity along the line shown in the merged image is graphed in the right panel. Scale bars represent 10 µm. (E, F) Fluorescence intensity signals of Rad51 (E) or EdU (F) at a minimum of 100 SV40 DNA replication centers from micrographs as in (D). Dashed and solid lines within boxes represent the average and median, respectively. Boxes contain the 25th–75th quartiles of intensities, whereas whiskers show minimum and maximum intensities.

We then verified that inhibition of ATM or ATR from 40 to 48 hpi produced similar aberrant DNA replication products to those previously reported when ATM or ATR were inhibited for a longer period (from 20 to 48 hpi Figures 1 and 2 and see reference [30]). Whole cell DNA extracted from SV40-infected BSC40 cells exposed to ATRi or ATMi from 40 to 48 hpi was digested with *Xba*I and *Sac*I, blotted, and probed for the SV40 genome. Similar to results obtained upon longer inhibitor treatment [30], viral DNA concatemer formation increased by 10 and 2.7 fold for ATMi and ATRi, respectively, compared to DMSO-treated infected cells (Figure 6A, left panel).

### ATM or ATR inhibition late in infection does not affect SV40 DNA replicative capacity in S phase but increases DNA-PK activation

The vigorous activation of ATM and ATR that accompanies viral DNA replication is required to prevent the appearance of aberrant viral DNA replication products that result from replication of broken viral DNA [30]. However, the mechanistic basis of this replication defect is not understood. Since ATM and ATR phosphorylate a plethora of targets including factors that influence HDR and NHEJ [57], [58], [59], we hypothesized that the viral replication defects associated with inactivation of ATM and ATR might correlate with changes in the activation or recruitment of DNA repair factors at the viral replication center. We first used immunoblotting to determine the phosphorylation state of residues on CHK1, CHK2, NBS1, and DNA-PK_cs_ in cell lysates from SV40- or mock-infected cells exposed from 40 to 48 hpi to DMSO, ATRi, or ATMi. The activation of DNA-PK_cs_, indicated by autophosphorylation of S2056, was increased upon ATRi or ATMi treatment relative to DMSO-treated SV40-infected cells (Figure 6B, compare lane 1 to lanes 2, 3). The presence of ATRi decreased the phosphorylation of both ATR and ATM substrates (Figure 6C, compare lane 1 to 2). We suggest that the effect of ATRi on phosphorylation of ATM substrates is an indirect consequence of the significantly reduced fraction of cells undergoing viral replication upon ATR inhibition (Figure S2B, C). ATM inhibition only mildly decreased the steady state levels of phosphorylated CHK2 and NBS1 (Figure 6C, compare lane 1 to lane 3). This modest response is not due to ineffectual repression of ATM activity since ATMi effectively blocks the phosphorylation of CHK2 in response to the DSB-inducing radiomimetic compound neocarzinostatin (Figure S3A, compare lane 2, 3 to 4, 5). Furthermore, ATM inhibition from 40 to 48 hpi decreased levels of the hyper-phosphorylated form of CtIP (Figure S3B, compare lane 1 to 2), an ATM and CDK dependent product [59]. Similar to observations in ATM-inhibited or ataxia-telangiectasia cells exposed to DNA damaging agents [60], these results suggest that ATR and DNA-PK_cs_ may cross-phosphorylate substrates of ATM during SV40 infection. Importantly, these results indicate that the brief 40 to 48 h inhibitor exposure is sufficient to produce the DNA-PK_cs_ activation previously observed with longer periods of ATM or ATR inhibition [30].

The accumulation of aberrant replication products observed when ATM or ATR were inhibited (Figure 6A–C) might correlate with a change in the distribution of repair proteins that associate with SV40 DNA replication centers. To examine whether DNA damage signaling during SV40 infection influences the association of DNA repair factors with viral DNA replication centers, the subcellular localization of several HDR and NHEJ proteins was assessed at 48 hpi by immunofluorescence microscopy of SV40- or mock-infected BSC40 cells exposed to DMSO, ATRi, or ATMi from 40 to 48 hpi. We first verified that treatment with the inhibitors did not interfere with bulk viral DNA replication at Tag foci. Importantly, replication centers are only present in S phase infected cells (Figure S2), and examination of the infected cell population that contains viral replication centers avoids the inclusion of the G1, G2, and M phase cell populations. ATRi-treated cells contain a large portion of non-S phase cells (Figure S2B, C) that decrease total viral DNA replication of ATRi-treated cells relative to DMSO-treated cells (Figures 1 and 6A [30]). Thus to test the general ability of ATRi- or ATMi-treated cells to replicate viral DNA in S phase, we examined the incorporation of EdU at the viral replication center.

As observed previously [27], [30], [61], chromatin-bound RAD51 and EdU incorporated during a 5 minute pulse administered immediately prior to fixation co-localized with Tag at 48 hpi in DMSO-treated, SV40-infected BSC40 cells (Figure 6D). Although the fraction of total cells exhibiting Tag staining decreased upon ATRi treatment (Figure S2C), in those cells containing viral replication foci, the EdU signal associated with Tag was more intense than in DMSO-treated, SV40-infected cells (Figure 6D, E). In contrast, treatment with ATMi led to a dispersion of Tag signal. However, EdU signal at these dispersed Tag foci was actually increased relative to the normal replication centers observed in DMSO-treated infected cells (Figure 6D, E).

To verify that EdU labeling in ATRi or ATMi treated cells was viral, not cellular, DNA replication, DNA was extracted from SV40- or mock-infected BSC40 cells treated with DMSO, ATRi, or ATMi from 40 to 48 hpi and pulsed with EdU for 5 minutes prior to extraction. DNA was cleaved with *Xba*I and *Sac*I, blotted, and probed with an antibody that cross-reacts with EdU (Figure 6A, right panel). The EdU-labeled DNA from SV40-infected cells treated with DMSO migrated as a unit-length supercoiled (I) and nicked (II) monomer, converging replication forks (LCI), dimers, and catenated intermediates (Figure 6A, right panel, lane 1), as expected for viral DNA. Replicating cellular DNA from uninfected cells migrated as a weaker smear of signal between 3 and 7 kilobases due to sensitivity of the cellular DNA to *Xba*I and *Sac*I endonucleases (Figure 6A, right panel, compare lane 1 to 4). EdU-labeled DNA from ATR inhibited cells migrated identically to that observed from the SV40-infected, DMSO-treated cells (Figure 6A, right panel, compare lane 1 to 2). EdU-labeled DNA from ATM-inhibited, SV40-infected BSC40 cells contained all the replication intermediates and products that occur in DMSO-treated SV40-infected cells, but also contained larger products and concatemers (Figure 6A, right panel, compare lanes 1, 4 to lane 3). We conclude that Tag and EdU staining in the presence of ATMi and ATRi mark replicating viral, not cellular, DNA.

RPA is actively loaded onto replicating single-stranded DNA by Tag during SV40 DNA replication [62]. Consistent with ongoing replication, the RPA subunit RPA70 colocalized with Tag when replication foci were observed in ATMi and ATRi-treated SV40-infected cells (Figure S3C). Relative to DMSO-treated SV40-infected cells, RPA70 intensity was unchanged upon ATRi treatment, but was slightly enhanced at the viral replication center when ATM was inhibited (Figure S3D). Independent of DSB formation during cellular DNA replication, single-stranded DNA, produced by helicase unwinding, is also bound by RAD51 [15], [20]. Similar to RPA, RAD51 remained co-localized with Tag regardless of whether ATM or ATR were inhibited (Figure 6D) with a minor, albeit significant, decrease in RAD51 fluorescence signal in both cases (Figure 6F). We conclude that RAD51 and RPA loading onto replicating single-stranded viral DNA are only mildly affected by ATR and ATM kinase activities.

### ATM inhibition decreases CtIP and BLM association with viral DNA replication foci

**Figure 7:**
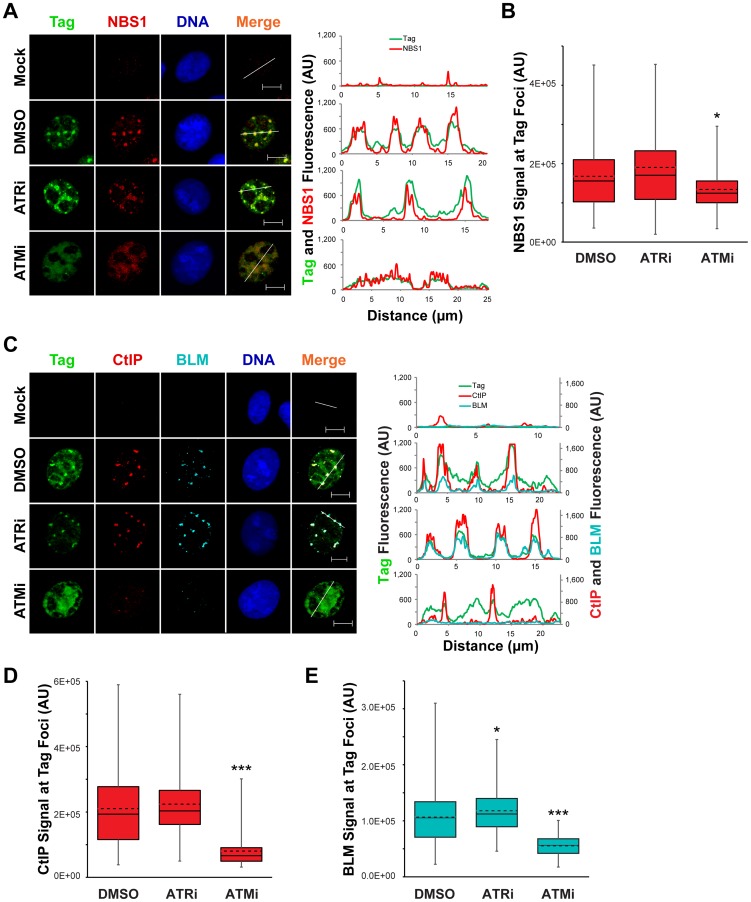
Localization of 5′ to 3′ DNA end resection proteins is promoted by ATM kinase. (A, C) Representative images at 48 hpi of ATRi-treated, ATMi-treated, or un-inhibited SV40-infected BSC40 cells probed for chromatin-bound Nbs1 and Tag (A) or CtIP, BLM, and Tag (C). DMSO, ATRi, or ATMi was present in the media from 40–48 hpi. Scale bars represent 10 µm. In the right panel, the fluorescence signals along the line in the merged image are graphed. (B, D, E) Nbs1 (B), CtIP (D), or BLM (E) fluorescence intensity signals at greater than or equal to 100 SV40 DNA replication centers from micrographs described in (A) and (C), respectively. The average and median are denoted by the dashed and solid lines, respectively. The 25th–75th quartiles of intensities are enclosed within the boxes. Minimum and maximum intensities are indicated by the whiskers.

The initiation of 5′ to 3′ end resection, an event required prior to strand invasion during HDR, has been implicated as a process that requires ATR- or ATM-dependent phosphorylations [59]. Therefore, we queried whether the enrichment of NBS1, CtIP, and BLM to SV40 replication centers requires ATM or ATR. The presence of ATRi from 40–48 hpi had no effect on the co-localization of Tag with NBS1, BLM, or CtIP compared to DMSO-treated, SV40-infected cells (Figure 7A, C). Likewise, NBS1 remained associated with Tag foci upon ATM inhibition with slightly decreased fluorescence intensity relative to DMSO- and ATRi-treated SV40-infected cells (Figure 7A, B). However both CtIP and BLM, proteins that function during end resection downstream of initial MRN recruitment [10], no longer colocalized with the majority of Tag when ATM was inhibited (Figure 7C). Additionally, the lack of co-localization with Tag coincided with a large decrease in the total fluorescence intensity of BLM and CtIP (Figure 7D, E). We conclude that ATM, but not ATR, inhibition results in a defect in CtIP and BLM recruitment to the viral replication center.

### ATM kinase activity prevents DNA-PK_cs_ activation at the viral replication center

**Figure 8:**
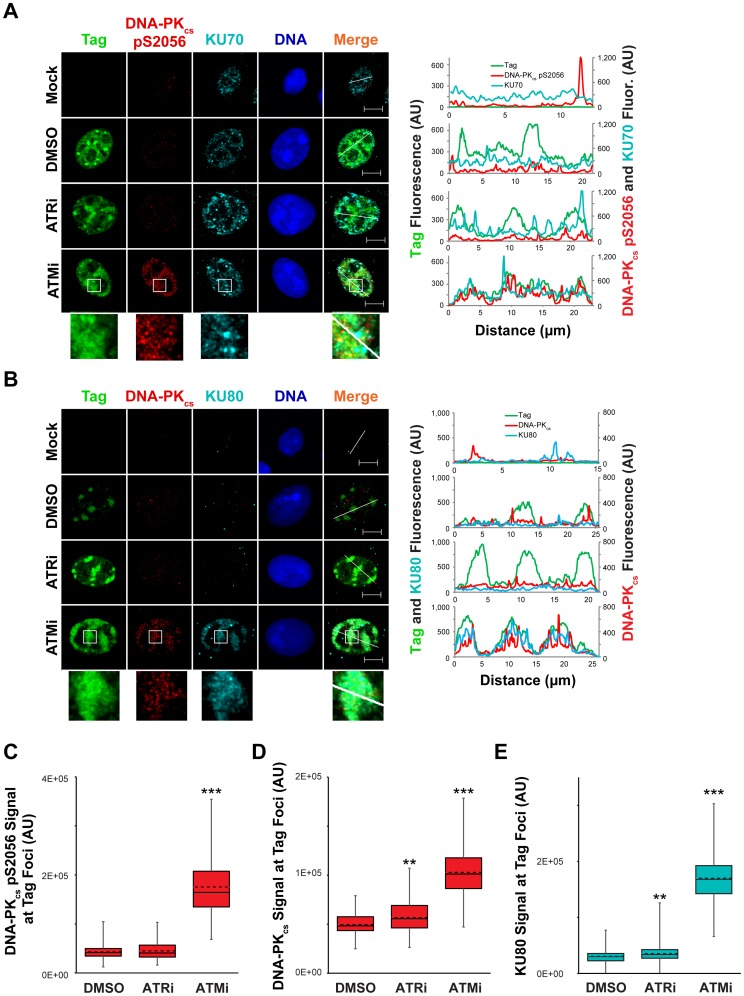
ATM kinase activity restricts DNA-PK localization and activation at SV40 Tag foci. (A, B) Micrographs of chromatin-bound NHEJ factors at 48 hpi from SV40-infected BSC40 cells treated with DMSO, ATRi, or ATMi from 40–48 hpi. Scale bars represent 10 µm. Fluorescence signals along the line in the merged imaged are graphed in the right panel. (C–E) DNA-PK_cs_ pS2056 (C), DNA-PK_cs_ (D), or KU80 (E) fluorescence intensities at a minimum of 70 (C) or 100 (D, E) SV40 DNA replication centers from images described in (A) and (B). The average and median are shown with dashed and solid lines, respectively. The boxes encompass the 25th–75th quartiles of intensities. Minimum and maximum intensities are portrayed by the whiskers.

DNA-PK_cs_ auto-phosphorylation is increased by ATM or ATR inhibition during viral DNA replication (Figure 6B and [30]). This change in the activation state of DNA-PK_cs_ could correspond to a difference in the localization of NHEJ factors to viral replication centers. This possibility was tested via immunostaining for chromatin-bound NHEJ components KU70, KU80, and DNA-PK_cs_ in SV40- or mock- infected BSC40 cells treated with ATRi, ATMi, or DMSO from 40 to 48 hpi prior to fixation. In DMSO-treated SV40-infected BSC40 cells, activated DNA-PK_cs_ (marked by S2056 phosphorylation) and KU70 were excluded from the Tag foci (Figure 8A). Inhibition of ATR from 40–48 hpi had no effect on the localization of KU70 or activated DNA-PK_cs_ (Figure 8A), consistent with the minor effect of ATRi on DNA-PK_cs_ activation (Figure 8B). In contrast, KU70 and activated DNA-PK_cs_ became prominent at the dispersed Tag replication centers following treatment with ATMi from 40–48 hpi (Figure 8A). The fluorescence signal intensity of DNA-PK_cs_ pS2056 was greatly enhanced upon ATMi treatment relative to DMSO or ATRi treatment (Figure 8C).

DNA-PK_cs_ and KU binding to DNA DSBs can be visualized by removing RNA from fixed cells with RNAseA [63]. This technique has been utilized to visualize exact sites of DSB formation in irradiated cells [63]. We employed RNAaseA pre-extraction of RNA-bound and non-chromatin bound protein on SV40-infected BSC40 cells exposed to DMSO, ATRi, or ATMi during the final 8 h of a 48 h SV40 infection. Similar to our previous extraction protocol, further RNAseA extraction of SV40-infected cells exposed to ATRi or DMSO revealed a complete lack of DNA-PK_cs_ and KU80 foci at Tag foci (Figure 8B). In contrast, ATM inhibition in cells infected with SV40 caused strong colocalization of DNA-PK_cs_ and KU80 with Tag foci (Figure 8B), accompanied by an increase in the florescence intensity of DNA-PK_cs_ and KU80 at the viral DNA replication center (Figure 8D, E). Taken together, these results indicate that ATM kinase activity prevents the recruitment of KU and DNA-PK activation at replicating viral DNA in Tag foci.

## Discussion

**Figure 9:**
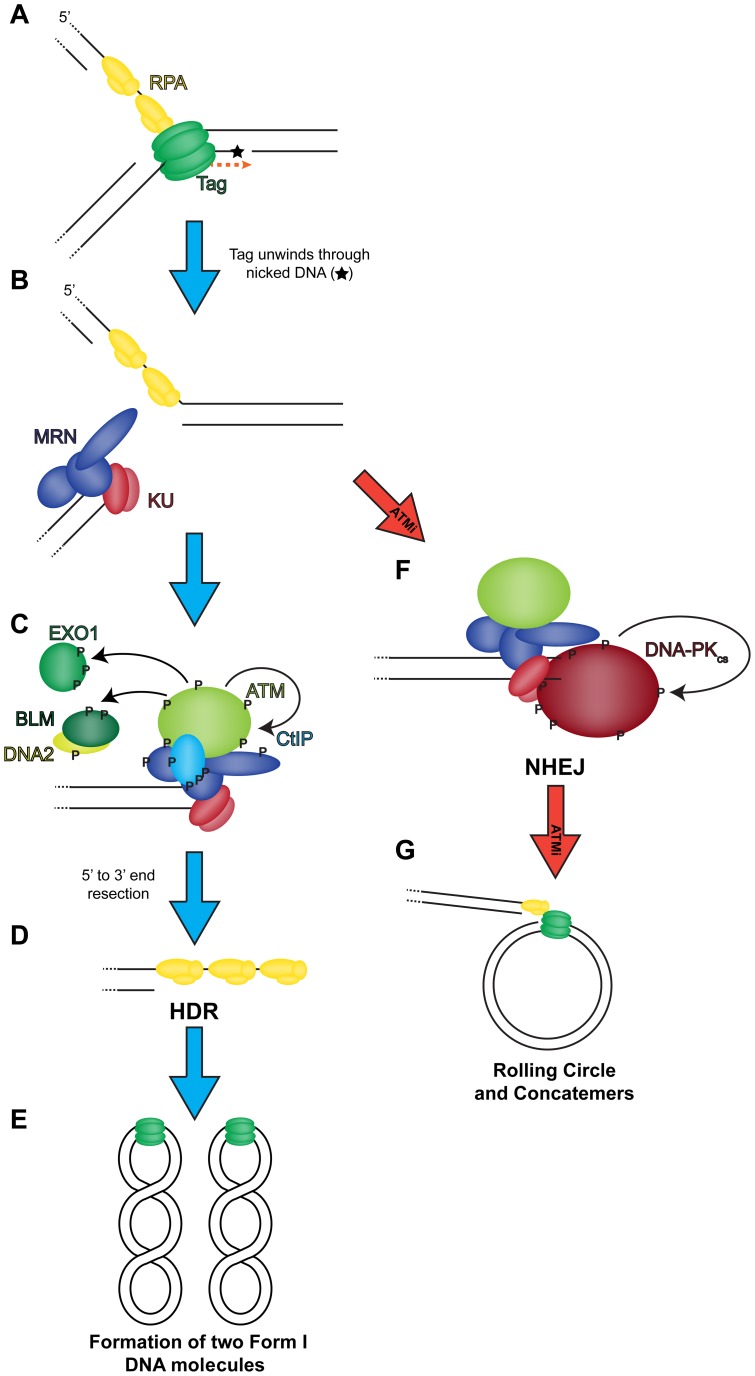
Model of the role of DNA double strand break repair during SV40 DNA replication. (A) Tag translocates toward a nick in the DNA (orange dashed arrow), unwinding the helix. (B) Helicase activity moves past the nick and generates a one-sided DSB that recruits MRN and KU to the DSB termini. Wild-type conditions (blue arrow): (C) MRN binding recruits and activates ATM at the DSB. ATM phosphorylates CtIP to create a stable interaction with NBS1 [59] at the DSB. ATM-phosphorylated MRN-CtIP catalyzes the initial 5′ to 3′ end resection of the DSB resulting in loss of KU. Further phosphorylation of BLM, DNA2, and EXO1 by ATM facilitates mobilization of each protein to broken DNA and more robust end resection leading to the loss of KU at viral DNA. (D) The single-stranded DNA generated by end resection is bound by RPA. Replacement of RPA on resected 3′ tail with RAD51 facilitates HDR, and the DSB is repaired to yield two intact unit length viral genomes at the end of the replication cycle (E). ATM inhibited conditions (red arrow): (F) MRN binding recruits ATM to DSB termini. However, failure of ATM to phosphorylate and create binding sites for CtIP, BLM/DNA2, or EXO1 causes stable KU binding to blunt or short single-stranded DNA at the DSB termini. Bound KU recruits DNA-PK_cs_ to the DSB. DNA-PK_cs_ kinase activity is activated to commence NHEJ. The DSB is not repaired efficiently by NHEJ resulting in rolling circle replication and concatemer formation (G).

Our data demonstrate that through the action of ATM kinase, SV40 recruits a complex, specific set of DNA repair activities to viral DNA replication centers that likely prevent accumulation of broken viral DNA. Evidence is presented that ATM kinase activity during viral DNA replication modulates the preference for HDR over NHEJ. Supporting the idea that ATR and DNA-PK_cs_ kinase activities aid in the repair of some breaks when ATM is inhibited, we found that inhibition of either ATR or DNA-PK in combination with ATM increased the amount of concatemers formed (Figure 1). However, only ATM and/or ATR inhibition, but not DNA-PK_cs_ inhibition, increased the appearance of long (∼20 kb) linear products (Figures 1 and 2), consistent with a role for ATM and ATR in preventing DSB formation or facilitating efficient DSB repair during SV40 chromatin replication. We demonstrate that SV40 replication centers colocalize with HDR factors (Figure 3) but do not associate with NHEJ proteins (Figures 4 and S1). Importantly, ATM- and ATR-dependent DNA damage signaling are both required for SV40-induced S phase arrest, but ATR kinase inhibition does not affect the stability of viral replication centers (Figures 5 and S2). Furthermore, we present data that ATM kinase activity is required for the colocalization of CtIP and BLM (but not RAD51, NBS1, and RPA) with Tag (Figures 6 and 7), implying that 5′ to 3′ end resection may be the primary aspect of HDR affected by ATM signaling. Although the retention of RPA and RAD51 appears inconsistent with the reduction of 5′ end resection factors [64], ATM and ATR inhibition from 40–48 hpi did not affect the ability of viral DNA to undergo replication as measured by EdU incorporation at the viral replication center (Figure 6). Therefore, the majority of RPA [62] and a portion of RAD51 [15], [20] at Tag foci are likely bound to replicating single-stranded DNA generated by Tag helicase activity. Inhibition of ATM kinase activity increased NHEJ factor association and DNA-PK_cs_ activation at the viral DNA replication center (Figures 8), indicating that ATM kinase activity prevents NHEJ activities from acting upon broken, replicating viral DNA. Collectively, our results are consistent with the hypothesis that ATM promotes viral DNA replication by controlling DSB repair pathway choice (Figure 9).

### ATM and ATR kinase activities contribute to DNA repair and cell cycle arrest during infection

ATM and ATR kinases have dynamic roles during SV40 infection in preventing DSB formation on replicating viral DNA (Figures 1, 2, and see reference [30]) and in activating CHK2 and CHK1, respectively, to potentiate S phase arrest (Figures 5 and S2). Our results imply that ATM and ATR kinases contribute differently to the S phase arrest occurring during SV40 infection (Figures 5 and S2) which helps to explain the differential effects on monomer production when ATM or ATR are inhibited (Figure 1 and reference [30]). Whereas ATM inhibition prior to 20 hpi decreased viral genome production [30] and increased the fraction of cells in G1 (Figure 5), the effect of ATMi treatment between 20 and 48 hpi on both cell cycle arrest and monomer production is relatively minor. In contrast, ATR kinase is needed throughout infection to enforce the S phase checkpoint (Figures 5 and S2) and, as a consequence, is required for efficient viral genome replication (Figure 1 and reference [30]). It is possible that the small number of replicating viral genomes present early during viral infection (0–20 hpi) [47], [48] provides insufficient replication stress or DNA breakage (Figure 2 and reference [65]) to elicit the ATR-CHK1 kinase activity required to arrest cells in S phase [66], [67]. Similarly, the amount of Tag present on chromatin during the early phase might be insufficient to deplete essential DNA replication factors away from host replisomes inside the infected cell [68] resulting in an inefficient ATR-CHK1 activation. The fact that ATM function is dispensable at later time points suggests that robust viral DNA replication activates ATR-CHK1 [30] sufficiently to maintain cell cycle arrest in S phase.

Prior to 20 hpi, the interplay of ATM-CHK2 and ATR-CHK1 kinase signaling during SV40 infection is reminiscent of that observed following ionizing radiation [69]. After a sufficient amount of ionizing radiation is administered during S phase [70], the ATM-CHK2 pathway is quickly activated to arrest the cell cycle, and DSB termini are processed to create 3′ tails. The 3′ tails subsequently serve as substrates for ATR kinase activation, and thereafter the ATR-CHK1 kinase pathway can maintain the checkpoint [69]. Such interplay may occur during SV40 infection as well when viral and cellular DNA suffers replication-associated breaks that efficiently activate ATM [27], [30]. Resection of these broken DNAs could activate ATR [69], providing for efficient S phase arrest of SV40 infected cells early during infection. Consistent with resection mediating ATR activation early during infection, ATM inhibition prior to 20 hpi decreased ATR activation whereas inhibition from 20 to 48 hpi did not affect ATR kinase function [30]. Notably, the proteins that CHK2 and CHK1 phosphorylate to arrest the cell cycle of infected cells are poorly understood. DNA damage signaling in uninfected cells during S phase arrests the cell cycle via CHK1 and CHK2-dependent phosphorylation of CDC25 family phosphatases [71], and other mechanisms have been suggested to medicate cell cycle arrest during SV40 infection [61]. We suggest that phosphorylation of CDC25 family phosphatases by CHK1 and CHK2 might represent a mechanism that contributes to cell cycle arrest during small DNA tumor virus infection.

### ATM activity promotes HDR and inhibits DNA-PK_cs_ activation at viral DNA replication centers

The large number of repair factors that associate with SV40 replication compartments was initially unexpected given the simplicity of the *in vitro* replication system [1]. However, cellular studies of other small DNA tumor viruses, including BKV [32], polyomavirus [72], MCV [73], JCV [31], [74], and HPV [29], have demonstrated that viral DNA replication can recruit numerous proteins that appear to fill roles that are dispensable *in vitro*. Prevention of DSB accumulation during viral DNA replication (Figures 1, 2, 9 and see reference [30]) appears to be the major function of the repair factors that accumulate at viral DNA replication foci. In spite of the collective activities of HDR, ATM, and ATR at replicating SV40 DNA, one ended DSBs in the form of rolling circles have been observed at low percentages in viral DNA extracted from unperturbed SV40-infected cells by both electron microscopy [47], [65] and 2D gel electrophoresis [30]. DSBs associated with cellular DNA replication are repaired by HDR [12]. Thus, the ATM-directed HDR activities at the viral replication center (Figures 3, 7) likely suppress the formation of rolling circles by promoting efficient repair of one-ended DSBs on replicating viral chromatin (Figure 9A–E). Conversely when ATM is inhibited, DNA-PK stably binds and is activated at DSB termini (Figure 8). In this circumstance, HDR fails to efficiently repair the replication-associated DSB, resulting in increased levels of rolling circles and linear viral products (Figures 1, 2, 6, 9F–G and reference [30]).

The prevention of DNA-PK recruitment to viral DNA replication centers and kinase activation during SV40 DNA replication is reminiscent of the effect of the cell cycle on NHEJ function [49], [75]. DNA-PK_cs_ kinase function is markedly decreased during S phase in response to several DNA-damaging agents [49]. This decrease in DNA-PK function results from the activation of 5′ to 3′ end resection during S phase [8], [9], [76], thereby resulting in an increased competition between MRN/CtIP and KU for binding to and processing of exposed ends of the DSB (Figure 9B). As both MRN/CtIP and KU possess activities that promote repair of DSBs by either HDR or NHEJ, respectively [58], binding one protein (e.g. KU) prior to the other (e.g., MRN/CtIP) might channel repair in one direction or another.

ATM is not required for homologous recombination [77], but its activity influences the repair kinetics of DSBs [78] and the extent of 5′ to 3′ end resection following treatment with exogenous DNA damaging agents [59], [75], [79]. Therefore, a plausible model for how ATM activity mediates DNA repair at viral DNA replication centers may be that ATM kinase activity activates or promotes stable association of 5′ to 3′ end resection proteins, including CtIP, EXO1, and BLM/DNA2 [10], with damaged viral DNA (Figures 7 and 9C). Importantly, ATM directly phosphorylates CtIP [59], EXO1 [80], and BLM [81], and ATM phosphorylation of CtIP influences the recruitment of BLM and EXO1, factors required for processive DSB end resection [10], to DSBs [59]. Such a mechanism might in turn prevent DNA-PK kinase activation by displacing KU from the DSB termini, allowing subsequent RAD51 binding to viral DNA and repair by HDR (Figure 9D, E).

In contrast, when ATM is inhibited during SV40 DNA replication, failure of ATM to phosphorylate CtIP, EXO1, and BLM/DNA2 might prevent processive end resection, resulting in inefficient digestion by chromatin-bound MRN at the viral DNA replication center [8] (Figure 7A, B, NBS1). By this model, KU then stably binds the one-ended replication-associated DSB (Figure 8). The KU-bound DSB can recruit DNA-PK_cs_, activate the DNA-PK_cs_ kinase (Figure 8), and create a potential barrier to efficient replication associated DSB repair (Figure 9F, G). The DNA-bound, active DNA-PK would search for a cognate DSB bound by another heterotrimer of DNA-PK. The kinetics of NHEJ at a replication-associated DSB are likely slow and inefficient, allowing rolling circle replication to generate numerous concatemers before DNA repair (Figures 8 and 9G). We suggest that the primary function of ATM at the SV40 DNA replication center is to promote 5′ to 3′ end resection of viral DNA. Determining how other proteins including ATR act to prevent DSB formation (Figure 1, 2) or promote DNA repair (Figure 9) during SV40 DNA replication will be an interesting endeavor. Future examination of repair pathways used during SV40 infection might reveal components of DNA repair or damage signaling that could be manipulated to prevent viral DNA replication during SV40 or other small DNA tumor virus infections.

## Materials and Methods

### Cells and SV40 infection

Complete Dulbecco's modified Eagle's medium (DMEM supplemented with 10% fetal bovine serum) was used to culture both BSC40 (ATCC) and U2OS (ATCC) cells. Plating for immunofluorescence and SV40 infection were as previously described [30].

### Plasmid transfection

For expression of HA-Topoisomerase IIIα and Flag-RMI2, 24 h prior to infection pCMV-HA-Topoisomerase IIIα or pIRES-neo-Flag-RMI2 [82] were transfected into BSC40 or U2OS cells using Fugene HD (Promega) using the manufacturer's protocol.

### DNA isolation, agarose gel electrophoresis, and Southern blotting

Total intracellular DNA was extracted from SV40- and mock-infected cells and subjected to agarose gel electrophoresis and Southern blotting as described [30]. In Figure 1, the same amount of SV40 DNA was loaded into each lane. In all other Southern blots, loading was normalized to cell number. In both cases, mitochondrial DNA was probed as an internal loading control. Loading determined by cell number or by mitochondrial DNA signal was highly correlated. 2D gel electrophoresis was as described by [30] with the following modification: the second dimension of the 2D gel was run for 7 h through a 0.95% 1×TBE agarose gel containing 0.5 ng/mL ethidium bromide. Southern blotting probes and data analysis using ImageQuant 5.2 were as previously described [30].

For DNA extractions from cells exposed to 20 µM EdU for 30 minutes (Figure 6A), DNA was isolated as per [30], but dissolved in 1 µL of 10 mM Tris pH 8.0 with 0.1 mM EDTA per 20,000 cells. A rat anti-BrdU antibody ([BU1/75 (ICR1)], Abcam), anti-rat conjugated to HRP (Jackson ImmunoResearch), and ECL-plus reagent (Perkin Elmer) were used to detect EdU label on the Southern blot as described in [83].

### Immunoblots

Cells were lysed as previously described [30]. The following antibodies that follow were used: anti-Tag (Pab101, [84]), anti-actin (I-19, Santa Cruz), anti-CHK1 (G-4, Santa Cruz), anti-CHK1 pS317 (Cell Signaling), anti-CHK2 pT68 (Y171, Epitomics), anti-CHK2 (EPR4325, Epitomics), anti-NBS1 pS343 (EP178, Epitomics), anti-NBS1 (A301-289A, Bethyl Laboratories), anti-ATR (N-19, Santa Cruz), anti-DNA-PK_cs_ (G-4, Santa Cruz), anti-KU80 (C-20, Santa Cruz), anti-KU70 (M-19, Santa Cruz), anti-DNA-PK_cs_ pS2056 (EPR5670, Epitomics), anti-CtIP (A300-488A, Bethyl Laboratories), and anti-GAPDH (0411, Santa Cruz).

### Immunofluorescence microscopy

Chromatin-associated proteins were visualized via pre-extraction of cells, prior to fixation and immunostaining as detailed by [26]. For RNAseA pre-extraction, 0.3 mg/mL RNAseA was included in pre-extraction buffer before fixation with 4% paraformaldehyde as previously described [63] and immunostaining was performed as before [30]. For EdU labeling of DNA, unless otherwise state, 20 µM EdU nucleoside in complete DMEM was added to cells for 5 minutes before fixation of cells and application of the click reaction at 48 hpi using the manufacturer's protocol (Invitrogen). An AxioObserver Z1 (Zeiss) equipped with a 63× Plan Apochromat (NA 1.4) oil objective (Zeiss) and an apotome (0.6 µm z slice) (Zeiss) was used to take all micrographs with two exceptions. For BLM and RMI1 in Figure 3A and B, a picture with a 1 µm z slice was taken using a LSM 510 META inverted microscope (Zeiss) equipped with a 63× Plan Apochromat (NA 1.4) oil objective (Zeiss).

To determine cell cycle phases of SV40-infected cells exposed to ATMi or ATRi (Figures 5C and S2B), the staining pattern of CENPF, EdU, and DAPI [56] in each cell was recorded from at least 300 cells. The average values were graphed in Figures 5C and S2B. Aberrant and normal Tag staining patterns from 3 independent experiments, each with at least 200 cells, were quantified from ATMi and ATRi treated cells and the average values were graphed in Figure 5D and tabulated in Figure S2C. Fluorescence intensity values at Tag focus were obtained using a 2.7 by 2.7 µm box in ImageJ. An equal area outside the nucleus near each Tag foci was used to obtain background intensity values. Fluorescence signal intensity values outside the replication center were subtracted from values at the viral replication center to correct for background. The corrected fluorescence intensities for a minimum of 70 viral replication centers are graphed in Figures 6 (E, F), 7 (B, D, E), 8 (C–E), and S3 (D).

Primary antibodies used for immunostaining were anti-Tag (Pab101), rabbit anti-Tag (in house), anti-CtIP (H-300, Santa Cruz), anti-CtIP (A300-488A, Bethyl Laboratories), anti-BRCA2 (ab-1, Calbiochem), anti-RAD52 (H-300, Santa Cruz), anti-BLM (C-18, Santa Cruz), anti-RMI1 (Novus), anti-Flag (M-2, Sigma), anti-HA (abcam), anti-CENPF (abcam), anti-DNA-PK_cs_ (G-4, Santa Cruz), anti-KU80 (C-20, Santa Cruz), anti-KU70 (M-19, Santa Cruz), anti-DNA-PK_cs_ pS2056 (EPR5670, Epitomics), anti-NBS1 (A301-289A, Bethyl Laboratories), anti-RAD51 (H-92, Santa Cruz), anti-RPA70B (In house), anti-PAR ([10H], Millipore), and anti-XRCC1 ([EPR4389(2)], abcam). Secondary antibodies used for immunostaining were anti-mouse conjugated to Alexa Fluor 488 (Invitrogen), anti-rabbit conjugated to Alexa Fluor 555 (Invitrogen), anti-goat conjugated to Alexa Fluor 555 (Invitrogen), and anti-goat conjugated to Alexa Fluor 647 (Jackson Immuno Research).

### Use of inhibitors

ATMi [36], ATRi [35], and Nu7026 [42] were as previously described [30] and used at 10, 5, and 10 µM, respectively. Olaparib [85] (AZD2281) (Selleck Chemicals) was dissolved in DMSO to 3 mM and used at a final concentration of 3 µM. An equal concentration of DMSO solvent was used as a control vehicle for each drug. Hydroxyurea (MP Biochemicals) was dissolved in water at a final concentration of 1 M and used at 0.5 and 10 mM in complete DMEM for 18 and 2 h, respectively.

To treat cells with inhibitors during the final 28 h or 8 h of a 48 h SV40 infection, the media was removed from the cells at 20 and 40 hpi, respectively, and replaced with fresh media containing either inhibitor or DMSO. Exposure of cells to inhibitors during periods of a 48 h SV40 infection was as per [30].

### Statistics

For Southern blotting data shown in Figure 1, the data analysis package of Microsoft Excel was used to perform statistics on appropriate data samples. A single factor ANOVA analysis was performed before t test. A two sample t-test assuming unequal variances was performed when the p<0.05 for the ANOVA test. In Figure 1, Bonferroni correction was applied when appropriate for multiple comparisons. One-tailed p values from t test are shown in Figure 1 (except for the ATRi to ATRi and Nu7026 t test which was two-tailed in Figure 1C). Unless otherwise indicated, bar graphs present the average of 3 or greater experiments and error bars represent standard deviation.

Comparisons of fluorescence intensity for Figures 6 (E, F), 7 (B, D, E), 8 (C–E), and S3B were performed using JMP 10.0.1. Normality and variance were evaluated by Shapiro-Wilk and Levene's test, respectively. Data that did not match a normal distribution were transformed. For data meeting the assumptions for a parametric test, single-factor ANOVA was used to test for a group effect. Significant ANOVA results (p<0.05) were followed by pair-wise comparisons by Tukey-Cramer. For data that violated the assumptions, a non-parametric Kruskal-Wallis test was used to test for a group effect. Significant Kruskal-Wallis results (p<0.05) were followed by pair-wise comparisons using the Mann-Whitney U test with a Bonferroni correction to allow for multiple comparisons.

p values are denoted by the number of asterisk(s): * p ≤ 0.05 ** p≤ 0.01 *** p≤ 0.001 **** p≤ 0.0001.

## Supporting Information

Figure S1 **NHEJ proteins are not localized to viral replication centers in SV40-infected U2OS cells.** (A, B) Representative images of chromatin-bound Tag or DNA-PK from SV40- or mock-infected U2OS cells at 48 hpi. Merged images show DNA-PK_cs_, Ku, and Tag. Bottom panel of (A) and (B) shows an enlargement of the region of the boxed area. Arrows point to an area on the line in which DNA-PK exclusion is more easily observed. The fluorescence intensity in arbitrary units (AU) along the line shown in the merged image is graphed in the right panel. Scale bars represent 10 µm.
(TIF)

Figure S2 **ATR inhibition increases cell cycling during SV40 infection in BSC40 cells.** (A) Scheme for treatment of SV40-infected cells with ATRi during a 48 h SV40 infection as described in Figure 5A. (B) Graph of the stage of the cell cycle of cells exposed to ATRi as in (A). Cell cycle phase was determined as described in Figure 5B. In (B), all G1 and S bars are significantly different (p<0.05 by two tailed student's t test) than the corresponding bars in DMSO or Mock controls except those denoted NS (not significant). The percent of cells in mitosis for the 40 to 48 hpi ATRi exposure is significantly different from both SV40- and mock-infected DMSO-treated controls (p<0.01 by two tailed student's t test). Error bars represent standard deviation. (C) Tabulated Tag staining patterns of SV40-infected cells exposed to DMSO, ATMi, or ATRi during the final 8 h of a 48 h infection. For EdU incorporation values, cells were exposed to EdU for 10 minutes prior to fixation. The presence of EdU in each population of cells with the indicated Tag staining pattern was determined by fluorescence microscopy for greater than 200 cells. Table shows the average of 3 independent experiments.
(TIF)

Figure S3 **RPA colocalizes with Tag foci independent of ATM and ATR signaling in SV40-infected BSC40 cells.** (A) Western blot of cell lysates extracted from BSC40 cells treated with the indicated amounts of NCS and DMSO/ATMi for 30 minutes. (B) Western blot of SV40- or mock-infected BSC40 cells exposed to DMSO or ATMi from 40 to 48 hpi. Lysates were prepared at 48 hpi. On NBS1 and CtIP blots, * denotes the hypophosphorylated band, and ** represents the hyperphosphorylated band. (C) Representative micrographs of chromatin-bound RPA70 at 48 hpi from SV40-infected BSC40 cells treated with DMSO, ATRi, or ATMi from 40–48 hpi. Scale bars represent 10 µm. The fluorescence signals along the line in the merged images are graphed in the right panel. (D) RPA70 fluorescence signal intensities at a minimum of 100 SV40 DNA replication centers from images described in (C). The average and median are shown with dashed and solid lines, respectively. The boxes encase the 25th–75th quartiles of intensities. Minimum and maximum intensities are shown by the whiskers.
(TIF)
