# Supplementary figures and images for: SV40 Utilizes ATM Kinase Activity to Prevent Non-homologous End Joining of Broken Viral DNA Replication Products
Source: PLoS Pathog. 2014 Dec 4;10(12):e1004536. doi: 10.1371/journal.ppat.1004536 (PMC4256475; doi:10.1371/journal.ppat.1004536)

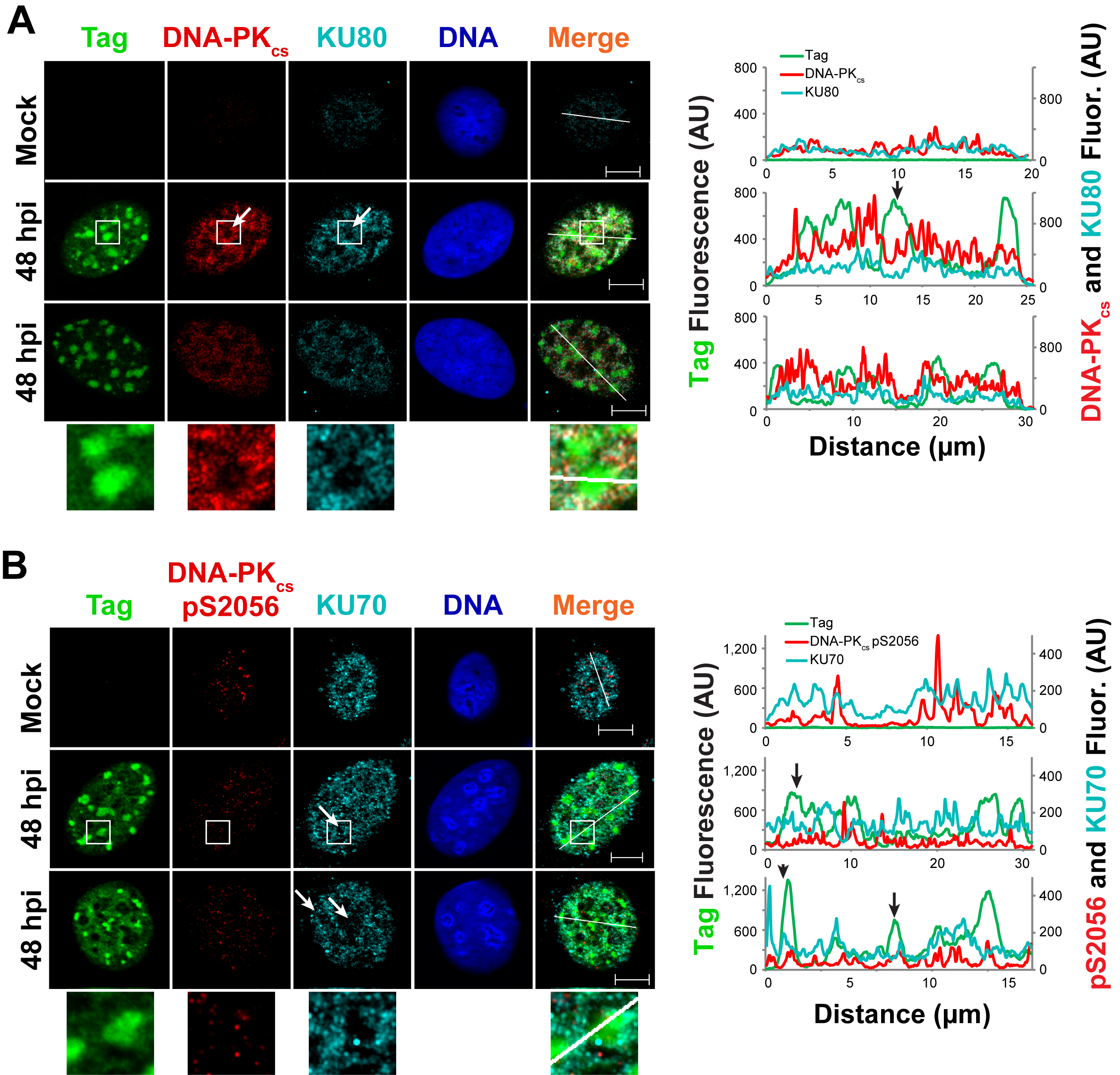

Supplement: Figure S1 — NHEJ proteins are not localized to viral replication centers in SV40-infected U2OS cells. (A, B) Representative images of chromatin-bound Tag or DNA-PK from SV40- or mock-infected U2OS cells at 48 hpi. Merged images show DNA-PKcs, Ku, and Tag. Bottom panel of (A) and (B) shows an enlargement of the region of the boxed area. Arrows point to an area on the line in which DNA-PK exclusion is more easily observed. The fluorescence intensity in arbitrary units (AU) along the line shown in the merged image is graphed in the right panel. Scale bars represent 10 µm. (TIF) [file ppat.1004536.s001.tif]

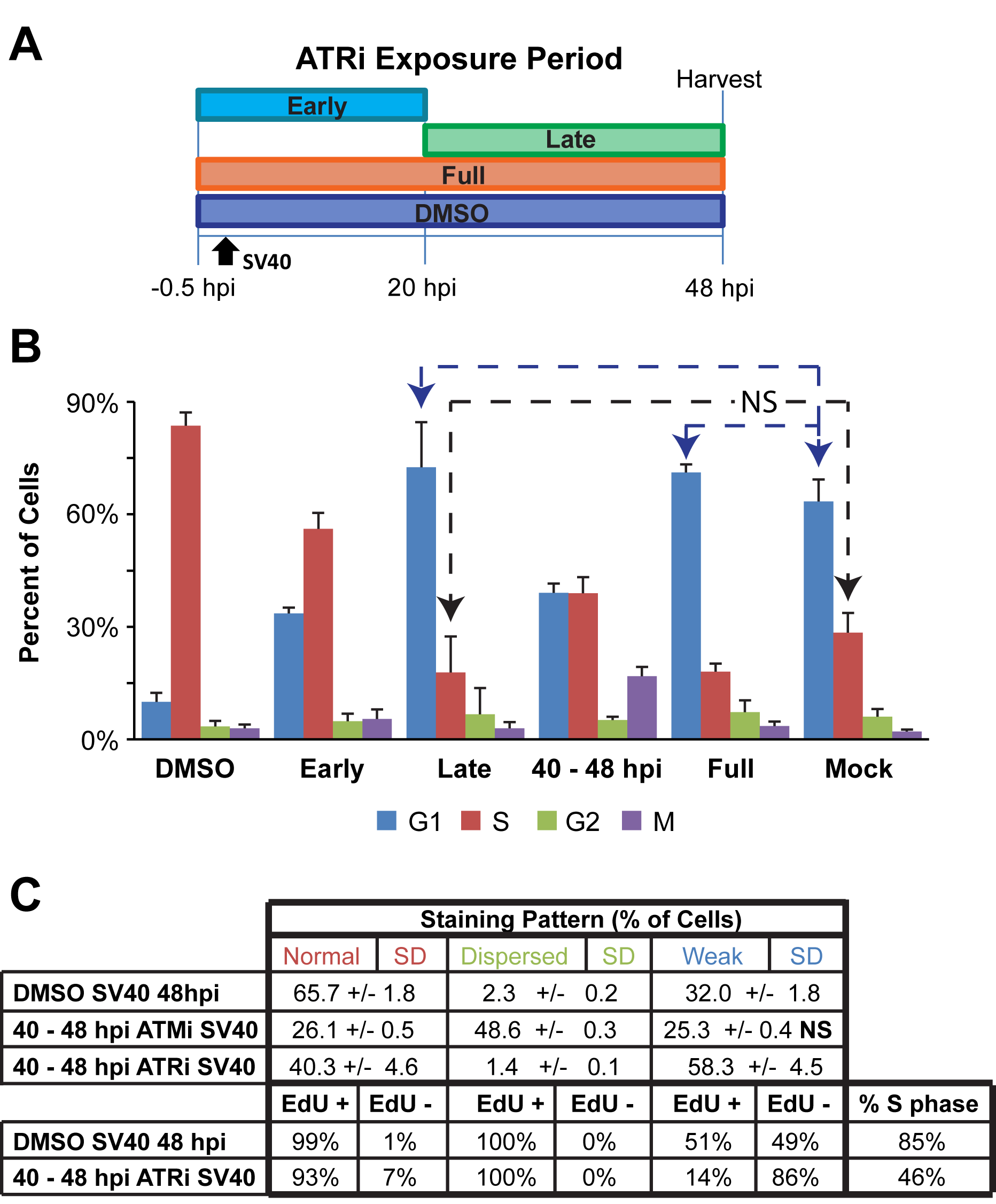

Supplement: Figure S2 — ATR inhibition increases cell cycling during SV40 infection in BSC40 cells. (A) Scheme for treatment of SV40-infected cells with ATRi during a 48 h SV40 infection as described in Figure 5A. (B) Graph of the stage of the cell cycle of cells exposed to ATRi as in (A). Cell cycle phase was determined as described in Figure 5B. In (B), all G1 and S bars are significantly different (p<0.05 by two tailed student's t test) than the corresponding bars in DMSO or Mock controls except those denoted NS (not significant). The percent of cells in mitosis for the 40 to 48 hpi ATRi exposure is significantly different from both SV40- and mock-infected DMSO-treated controls (p<0.01 by two tailed student's t test). Error bars represent standard deviation. (C) Tabulated Tag staining patterns of SV40-infected cells exposed to DMSO, ATMi, or ATRi during the final 8 h of a 48 h infection. For EdU incorporation values, cells were exposed to EdU for 10 minutes prior to fixation. The presence of EdU in each population of cells with the indicated Tag staining pattern was determined by fluorescence microscopy for greater than 200 cells. Table shows the average of 3 independent experiments. (TIF) [file ppat.1004536.s002.tif]

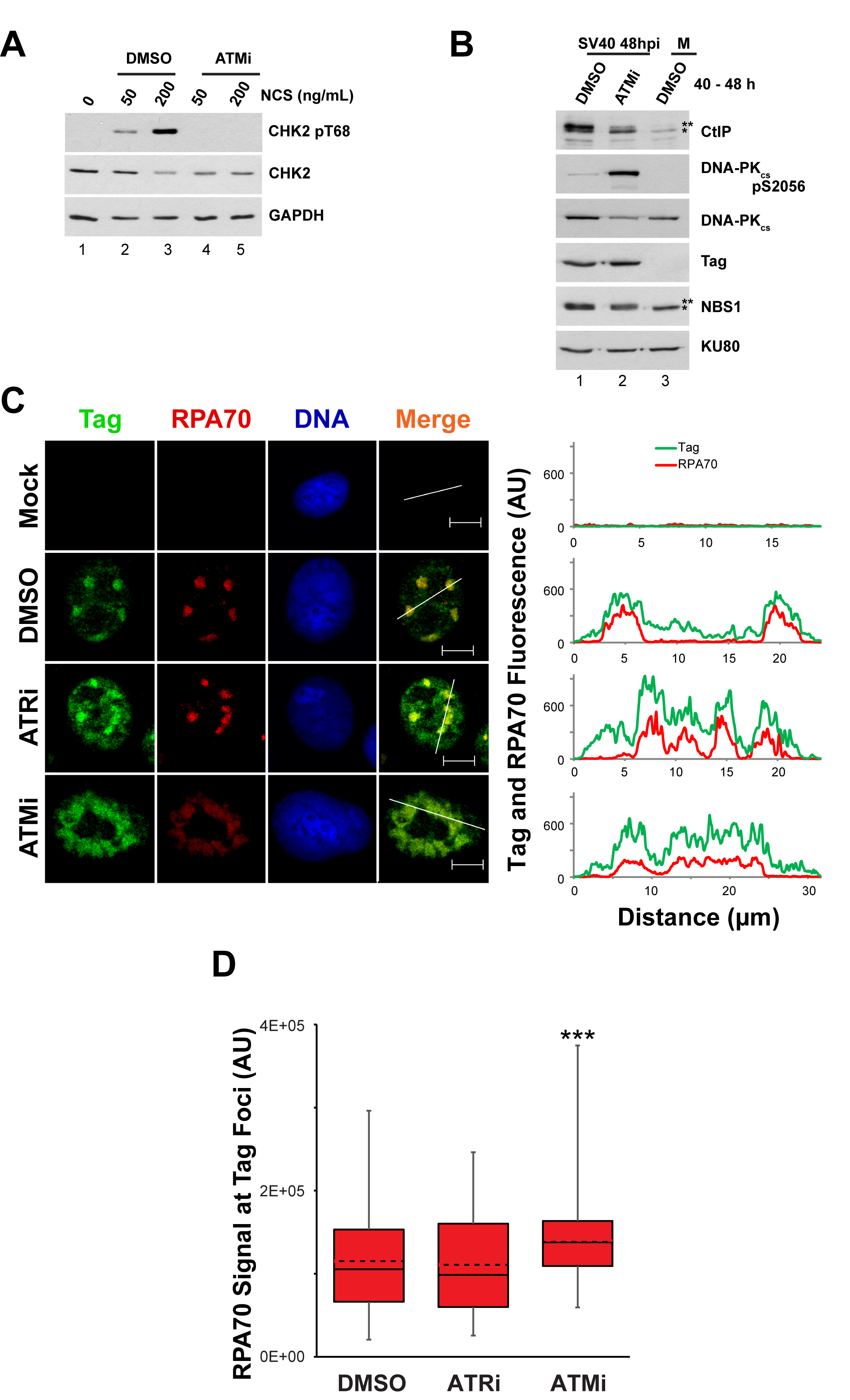

Supplement: Figure S3 — RPA colocalizes with Tag foci independent of ATM and ATR signaling in SV40-infected BSC40 cells. (A) Western blot of cell lysates extracted from BSC40 cells treated with the indicated amounts of NCS and DMSO/ATMi for 30 minutes. (B) Western blot of SV40- or mock-infected BSC40 cells exposed to DMSO or ATMi from 40 to 48 hpi. Lysates were prepared at 48 hpi. On NBS1 and CtIP blots, * denotes the hypophosphorylated band, and ** represents the hyperphosphorylated band. (C) Representative micrographs of chromatin-bound RPA70 at 48 hpi from SV40-infected BSC40 cells treated with DMSO, ATRi, or ATMi from 40–48 hpi. Scale bars represent 10 µm. The fluorescence signals along the line in the merged images are graphed in the right panel. (D) RPA70 fluorescence signal intensities at a minimum of 100 SV40 DNA replication centers from images described in (C). The average and median are shown with dashed and solid lines, respectively. The boxes encase the 25th–75th quartiles of intensities. Minimum and maximum intensities are shown by the whiskers. (TIF) [file ppat.1004536.s003.tif]
